# Biclustering of gene expression data by non-smooth non-negative matrix factorization

**DOI:** 10.1186/1471-2105-7-78

**Published:** 2006-02-17

**Authors:** Pedro Carmona-Saez, Roberto D Pascual-Marqui, F Tirado, Jose M Carazo, Alberto Pascual-Montano

**Affiliations:** 1 BioComputing Unit. National Center of Biotechnology. Campus Universidad Autónoma de Madrid. 28049. Spain; 2 The KEY Institute for Brain-Mind Research, University Hospital of Psychiatry. Lenggstr. 31, CH-8029 Zurich, Switzerland; 3 Computer Architecture Department. Facultad de Ciencias Físicas. Universidad Complutense de Madrid. 28040. Spain

## Abstract

**Background:**

The extended use of microarray technologies has enabled the generation and accumulation of gene expression datasets that contain expression levels of thousands of genes across tens or hundreds of different experimental conditions. One of the major challenges in the analysis of such datasets is to discover local structures composed by sets of genes that show coherent expression patterns across subsets of experimental conditions. These patterns may provide clues about the main biological processes associated to different physiological states.

**Results:**

In this work we present a methodology able to cluster genes and conditions highly related in sub-portions of the data. Our approach is based on a new data mining technique, Non-smooth Non-Negative Matrix Factorization (*n*sNMF), able to identify localized patterns in large datasets. We assessed the potential of this methodology analyzing several synthetic datasets as well as two large and heterogeneous sets of gene expression profiles. In all cases the method was able to identify localized features related to sets of genes that show consistent expression patterns across subsets of experimental conditions. The uncovered structures showed a clear biological meaning in terms of relationships among functional annotations of genes and the phenotypes or physiological states of the associated conditions.

**Conclusion:**

The proposed approach can be a useful tool to analyze large and heterogeneous gene expression datasets. The method is able to identify complex relationships among genes and conditions that are difficult to identify by standard clustering algorithms.

## Background

DNA microarray technology is a powerful method for monitoring the expression level of thousands of genes, or even whole genomes, in a single experiment. In the last few years, this technique has been widely used in several contexts such as tumor profiling, drug discovery or temporal analysis of cell behavior (for a review see [1]). Due to the widespread use of this high-throughput technique in the study of several biological systems, a large collection of gene expression datasets is available to the scientific community, some of which contain tens or hundreds of different experimental conditions and constitute reference databases or "compendiums" of gene expression profiles (see for example [2-6]).

One of the main goals in the analysis of such datasets is to identify groups of genes, or groups experimental conditions, that exhibit similar expression patterns. Several clustering techniques, such as k-means [7], self-organizing maps (SOM) [8,9] or hierarchical clustering [10] have been extensively applied to identify groups of similarly expressed genes or conditions from gene expression data. Additionally, hierarchical clustering algorithms have been also used to perform two-way clustering analysis in order to discover sets of genes similarly expressed in subsets of experimental conditions by performing clustering on both, genes and conditions, separately (some examples can be found in [3,4,11-13]). The identification of these block-structures plays a key role to get insights into the biological mechanisms associated to different physiological states as well as to define gene expression signatures, *i.e*., "genes that are coordinately expressed in samples related by some identifiable criterion such as cell type, differentiation state, or signaling response" [13].

Although standard clustering algorithms have been successfully applied in many contexts, they suffer from two well known limitations that are especially evident in the analysis of large and heterogeneous collections of gene expression data:

i) They group genes (or conditions) based on global similarities in their expression profiles. However, a set of co-regulated genes might only be co-expressed in a subset of experimental conditions, and show not related, and almost independent expression patterns in the rest. In the same way, related experiments may be characterized by only a small subset of coordinately expressed genes. Indeed, as Wang *et al*. remarked, there may only be a few gene components that account for most of the response variation across experiments, and thus important relationships among them may be lost in a high dimensional gene space [14].

ii) Standard clustering algorithms generally assign each gene to a single cluster. Nevertheless, many genes can be involved in different biological processes depending on the cellular requirements and, therefore, they might be co-expressed with different groups of genes under different experimental conditions [15]. Clustering the genes into one and only one group might mask the interrelationships between genes that are assigned to different clusters but show local similarities in their expression patterns.

In the last few years several methods have been proposed to avoid these drawbacks [15-18]. Among these methods, biclustering algorithms have been presented as an alternative approach to standard clustering techniques to identify local structures from gene expression datasets. These methods perform clustering on genes and conditions simultaneously in order to identify subsets of genes that show similar expression patterns across specific subsets of experimental conditions and vice versa. For an overview of biclustering methods see the revision of Madeira and Oliveira [19] and Tanay *et al*. [20].

A particularly promising technique, Non-Negative Matrix Factorization (NMF), has been recently introduced to the analysis of gene expression data in two independent works [21,22]. NMF can be applied to reduce the dimensionality of the data yielding a representation of conditions as a linear combination of a reduced set of *k*-factors. In this context, the factors represent sets of genes that behave in a strongly correlated fashion in sub-portions of the data. Kim and Tidor used this method to cluster genes based on local patterns and predict functional relationships in yeast while Brunet *et al*. focused their work on the analysis of samples projected in the reduced space, showing the usefulness of this approach for finding non-overlapping partitions of tumor samples.

In this paper, we present an extension of this technique to the analysis of gene expression data in a two-dimensional context, simultaneously clustering genes and conditions highly related in sub-portions of the data. The main purpose of this work is to show the potential of this method to identify gene expression modules, *i.e*. sets of genes that share local similarities in their expression patterns, as well as to identify the experimental conditions highly associated to these modules. We have used a new variant of the classical NMF model, the Non-smooth Non Negative Matrix Factorization algorithm (*ns*NMF) [23], that it has been specially developed to produce sparse representation of the factors and encoding vectors by making use of non-smoothness constraints. The sparseness introduced by this algorithm produce more compact and localized feature representation of the data than standard NMF, as it will be presented throughout this work.

To assess the potential of our approach and to illustrate its functionality we applied it to the analysis of synthetic data as well as two large and heterogeneous gene expression datasets, one comprising expression levels for thousands of genes across a large set of diverse human tissues [6] and the other containing gene expression profiles of several soft-tissue tumor types [24]. In all cases, the approach we propose was able to cluster sets of genes and conditions that were related in sub-parts of the data. The analysis of functional annotations that were significantly over-represented in each gene module provided meaningful insights about the biological events associated to the experimental conditions. Additionally, our methodology was able to find complex and less natural patterns that could not be detected by standard clustering algorithms. Additional material and figures are available at the website [25].

## Results

The goal of this study is to determine whether the proposed methodology is able to uncover local structures from gene expression data. To this end, we have used several simulated datasets containing different types of embedded structures as well as two large and heterogeneous gene expression datasets. In the next sections we describe an overview of the methodology and the detailed results of its application to the analysis of the synthetic and real datasets.

### General model for discovering local structures by *ns*NMF

Non-smooth non negative matrix factorization, as well as the classical NMF model, can be used to approximately reproduce a gene expression matrix **V **of dimension *m *genes and *n *samples as a product of two matrices **W **and **H**, with dimensions *mxk *and *kxn *respectively, where *k *<<*m*. The *k *columns of **W **have the dimension of a single array (*m *genes) and are known as factors or "basis experiments". The columns of **H **are known as encoding vectors and are in one-to-one correspondence with a single experiment of the gene expression data matrix (matrix **V**). Therefore, each row of **H **has the dimension of a single gene (*n *experiments) and it is denoted as "basis gene".

Each factor or basis experiment yielded by *ns*NMF contains a relatively small set of genes with non-zero coefficients that determine a local gene expression feature. These genes behave in a strongly correlated fashion in a sub-portion of the data and constitute a gene module. In the same way, coefficients in basis genes are used to determine the set of experimental conditions highly associated to these modules. In other words, the set of genes and experimental conditions that show high values in the same basis experiment (*lth *column of **W**) and its corresponding basis gene (*lth *row of **H**) respectively are highly related in only a sub-portion of the data and constitute a gene expression bicluster.

**Figure 1:**
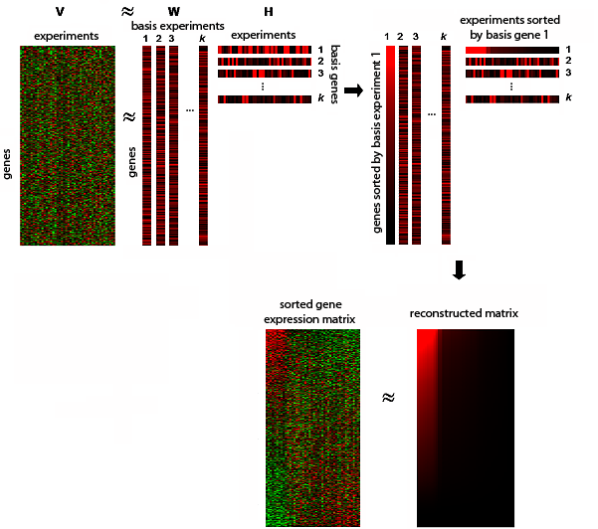
**General schema of the method ***ns*NMF approximates the original matrix as a product of two submatrices, **W **and **H**. Columns of **W **are basis experiments while rows of **H **constitute basis genes (columns of **W **and rows of **H **are separated for a better visibility). Coefficients in each pair of basis gene and experiment are used to sort conditions and genes in the original matrix. Conditions and genes with high values in the same basis gene and basis experiment are highly related in a sub-portion of the data and are co-clustered in the upper left corner of the sorted array.

Figure 1 shows the general schema of our approach. Given a certain factor, *i.e*., the *lth *column of **W**, all genes in the dataset can be properly sorted by their association to the local pattern captured by this factor. At the same time, conditions can also be sorted by their coefficients in the corresponding basis gene, that is, the *lth *row of **H**. This operation is carried out in one-to-one correspondence among columns of **W **and rows of **H**, generating *k *natural ordinations of the gene expression matrix in which genes and experiments highly related in a sub-portion of the data are placed in the upper left corner of the array.

Due to differences in the initial conditions used in the factorization this procedure can generate different sets of results across different runs of the algorithm. In this work we have exploited this non-deterministic nature of the *ns*NMF algorithm to determine the consistency of the uncovered patterns in two different ways. First, we have used the model selection method proposed by Brunet *et al*. [21] to determine the number of factors associated to stable partitions of conditions. Second, we have evaluated the consistency of the gene modules obtained at a given rank by selecting the set of genes that were more representative in each factor and evaluating their consistency across several runs of the algorithm (see Methods).

### Synthetic data

**Figure 2:**
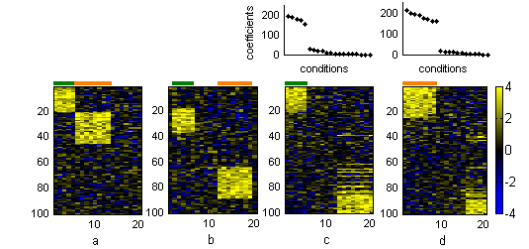
**Results from synthetic dataset A **(a) Original dataset with the two embedded patterns. (b) Dataset sorted by two-way hierarchical clustering. Dataset sorted by (c) the first basis gene and basis experiment and (d) the second basis gene and basis experiment yielded by *ns*NMF at *k *= 3. Conditions belonging to pattern Pla are marked in green and conditions belonging to pattern P2a are depicted in orange. The two plots over the heatmaps represent the coefficients of conditions in each sorted basis gene.

In order to test the potential of the method and its relative performance with respect to standard two-way hierarchical clustering analysis we first applied it to the analysis of two synthetic datasets containing overlapping and non-overlapping structures. In the first dataset (dataset A), we aimed at testing the capacity of *ns*NMF in extracting non-overlapping obvious structures. This dataset contains two block structures embedded into a 100 × 20 noisy matrix. Figure 2 depicts the structures Pla (20 rows and 5 columns) and P2a (25 rows and 8 columns). In the rest of this document we will refer to rows as genes and columns as conditions. As it was expected, average linkage two-way hierarchical clustering correctly grouped together genes and conditions belonging to both structures. Based on the cluster dendrogram, conditions can be clearly separated into two groups, each one containing the set of conditions belonging to each one of the embedded patterns and some conditions related to background noise. Nevertheless, the cluster dendrogram does not seem to suggest a clear sub-structure of three or more classes (see additional file 1, figure 1).

We then applied *ns*NMF to this data set. Based on the cophenetic correlation coefficient we found high stability of the model at ranks 2 and 3 (see additional file 1, figure 2). Matrix factorization at *k *= 3 was able to correctly partition the set of genes and conditions belonging to each one of the embedded patterns. When the matrix was sorted by the values of the first basis experiment and the corresponding basis gene the set of genes and conditions belonging to Pla were grouped together in upper-left corner of the array. In the same way, genes and conditions belonging to P2a were co-clustered by the second basis experiment and basis gene (see figure 2). As it was expected, only gene modules defined by these two factors showed a high consistency across different runs of the algorithm (these modules were repeated in 100% of the factorizations), while genes belonging to the gene module defined by the third factor, related to background noise, varied across different factorizations (was only found in around 60% of the factorizations).

**Figure 3:**
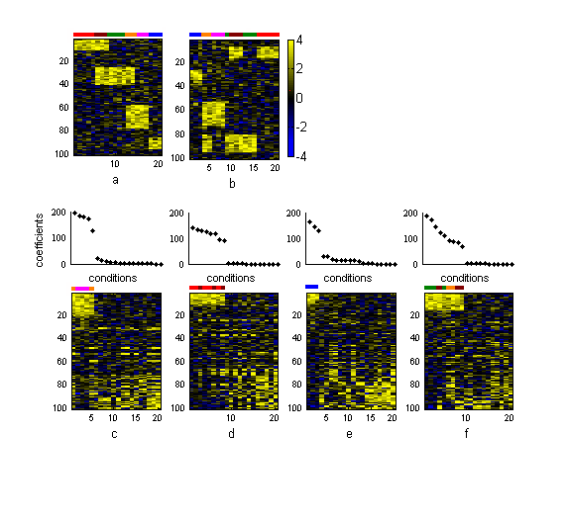
**Results from synthetic dataset B **(a) Original dataset with the three embedded patterns and (b) the same dataset sorted by two-way hierarchical clustering. Heatmaps of the original dataset sorted by the (c) first, (d) second, (e) third and (f) fourth basis genes and basis experiments yielded by *ns*NMF at *k *= 4 are shown in the bottom part of the figure. Non-overlapping conditions of Plb are marked in red, non-overlapping conditions of P2b are marked in green and non-overlapping conditions of P3b are marked in magenta. The overlapped area between Plb and P2b is marked in brown while the overlapped columns between P2b and P3b are marked in orange. Columns of P4b are marked in blue. Plots over the heatmaps represent coefficients of conditions in each sorted basis gene. The sorted basis genes present gaps indicating the set of conditions belonging to each pattern.

We also tested the potential of our method to identify overlapped structures. Figure 3 shows the results obtained in the analysis of dataset B, which contains four embedded patterns Plb, P2b, P3b and P4b of sizes 10 × 8, 15 × 9, 20 × 5 and 10 × 3 respectively. In this dataset P2b shares three columns with Plb and two columns with P3b. Two-way hierarchical clustering performed well at grouping conditions and genes belonging to P4b while did not succeed in correctly identifying overlapped structures. Conditions that overlap their expression profiles with different sets of conditions were associated to only one cluster. For example, columns that were overlapped among Plb and P2b (marked in brown in figure 3) were co-clustered with non-overlapping columns of P2b (marked in green) but not with non-overlapping columns of Plb (marked in red). This was not surprising because this algorithm, as well as other classical clustering techniques, groups objects into discrete clusters masking potential relationships among objects grouped into different clusters.

Based on the cophenetic correlation coefficient we could attest the robustness of the model at *k *= 4. In addition, the gene modules defined by the four factors were highly consistent and they were all found in more than 95% of the factorizations. As can be seen in figure 3, when the genes and the samples were sorted by their values in each basis experiment and basis gene obtained in the factorization, the four embedded patterns were correctly identified, including P2b that overlapped with two different structures. The first basis gene and basis experiment grouped together conditions and genes belonging to P3b, the second identified Plb, the third identified P4b and, finally, the fourth basis gene and basis experiment co-clustered conditions and genes belonging to P2b.

As we have mentioned previously, even if NMF has been presented and used as a method capable of finding the underlying parts-based structure of complex data, there is no explicit guarantee in the method to support this property. This was the main motivation to develop and use a new matrix factorization technique capable of producing more localized, less smooth feature representations of the data like the *ns*NMF model. The sparsification on both the factors (**W**) and the encodings (**H**) tends to decrease the relevance of the non-significant elements in each feature, while reinforcing at the same time the most relevant ones. This fact was illustrated by applying standard NMF and *ns*NMF to the analysis of dataset A (see additional file 1, figure 4). This comparative analysis shows that basis experiments and basis genes obtained by NMF are not really sparse while those yielded by *ns*NMF represented more compact features of the dataset. In this way, values of non-relevant conditions and genes in each local pattern were significantly reduced in the case of *ns*NMF, which is more pronounced when the sparseness parameter is increased. This sparsification procedure is, therefore, intrinsic to the structure of the data. Similar results were obtained when standard NMF and *ns*NMF were applied to real gene expression data (see additional file 1, figures 5, 6 and 7), in which *ns*NMF generated more compact local features than the standard NMF method.

**Figure 4:**
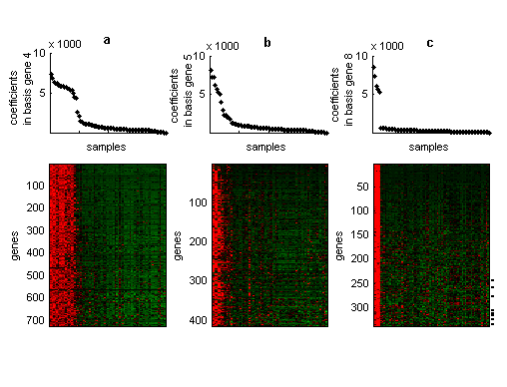
**Structures from the human transcriptome dataset **Plots in the first row represent coefficients of samples in the (a) fourth, (b) fifth and (c) eighth sorted basis genes. Heatmaps in the second row represent the expression matrix in which genes (in rows) and samples (in columns) are sorted by their coefficients in the corresponding basis experiment and basis gene. Only genes that were highly representative of each basis experiment are shown. Dash lines in the third heatmap represent positions of genes that were included in the testis-gene module but were clustered in distant positions to the testis-gene group by hierarchical clustering.

**Figure 5:**
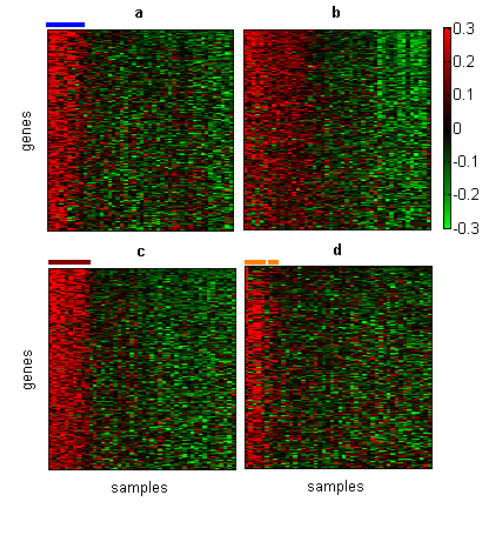
**Structures from the soft-tissue tumor dataset **Each heatmap represents the expression matrix in which samples and genes were sorted by (a) the first, (b) second, (c) third and (d) fourth basis gene and basis experiment. Only genes that were selected as highly representative of each basis experiment are shown. Blue line corresponds to monophasic synovial sarcomas, brown line to gastrointestinal stromal tumors and orange line to six of the eleven leiomyosarcomas samples.

### Human transcriptome dataset

This dataset contains expression levels of thousands of human genes across a panel of 79 human tissues and is an invaluable source of information for the analysis of the human transcriptome [6]. Due to its size and heterogeneity, this dataset is an ideal target to identify local structures of gene expression. Uncovering and linking gene expression modules to phenotypic variation of cells or tissues can provide clues about tissue-specific functions or the molecular organization of diverse cells.

We applied *ns*NMF with a rank value of 8, value in which the model showed a marked robustness as was attested by the corresponding peak in the cophenetic correlation coefficient. Three of the eight gene expression modules, those corresponding to factors 4, 5 and 8, showed a noticeable consistency (were found in more than 80% of the factorizations) and the structures related to these modules were further examined.

The fourth basis experiment clustered a set of genes mainly expressed in samples from neural and brain tissues (tissues from amygdala, prefrontal cortex, occipital lobe, whole brain, cingulated cortex, caudate nucleus, cerebellum, parietal lobe, subthalamic nucleus, medulla oblongata, globus pallidus, fetal brain, thalamus, cerebellum peduncles, hypothalamus, temporal lobe, pons, spinal cord, pituitary and olfactory bulb) which were clearly partitioned by the corresponding fourth basis gene (figure 4a). Several GO annotations related to neural functions such as "neurogenesis", "synaptic transmission" or "central nervous system development" were significantly over-represented in this module (see table 1a), which concord with the phenotype of samples that were highly associated to this module.

**Table 1:** Enrichment of GO categories in gene modules. Enrichment of GO categories in gene modules obtained from (a) the human transcriptome dataset and (b) the soft tissue tumor dataset. Only functional categories containing at least 6 genes and *p*-values less than 0.01 are reported.

a)			
**Factor**	**biological process**	**#genes**	**p-value**
Factor 4 (726 genes)	Neurogenesis	43	0.0
	Cell adhesion	33	1.30E-04
	Transport	32	0.003
	Synaptic transmission	31	0.0
	Regulation of transcription, DNA-dependent	25	0.0
	Central nervous system development	17	0.0
	Small GTPase mediated signal transduction	17	4.70E-04
	Potassium ion transport	11	0.008
	Sodium ion transport	10	2.40E-04
	Microtubule-based movement	9	0.0
	Neuropeptide signaling pathway	8	2.40E-04
	Regulation of apoptosis	8	0.009
	ATP synthesis coupled proton transport	6	0.001
	Microtubule polymerization	6	3.00E-05
	Vesicle-mediated transport	6	7.70E-04
Factor 5 (414 genes)	Immune response	78	0.0
	Signal transduction	47	0.0
	Intracellular signaling cascade	29	0.0
	Inflammatory response	26	0.0
	Cellular defense response	21	0.0
	Antigen presentation, endogenous antigen	18	0.0
	Antigen processing, endogenous antigen via MHC class I	18	0.0
	Proteolysis and peptidolysis	17	0.003
	Cell motility	16	0.0
	Cell surface receptor linked signal transduction	15	0.0
	Chemotaxis	13	0.0
	Positive regulation of l-kappaB kinase/NF-kappaB cascade	13	0.0
	Regulation of apoptosis	12	0.0
	Heterophilic cell adhesion	11	0.0
	Antimicrobial tumoral response (sensu Vertebrata)	10	1.00E-05
	Small GTPase mediated signal transduction	10	0.004
	Anti-apoptosis	9	1.40E-04
	Defense response	8	7.00E-05
	Induction of apoptosis	8	0.002
	Response to virus	7	0.0
	Cell recognition	6	0.0
	Integrin-mediated signaling pathway	6	2.90E-04
Factor 8 (339 genes)	Spermatogenesis	13	0.0
	Transcription	11	0.0
	Mitosis	6	0.002


b)			
**Factor**	**biological process**	**#genes**	**p-value**
Factor 1 (546 genes)	Regulation of transcription, DNA-dependent	32	2.00E-05
	Development	16	0.003
	Neurogenesis	9	0.008
	Transcription from Pol II promoter	7	0.007
	Morphogenesis	6	0.002
	Skeletal development	6	0.007
	Chromosome organization and biogenesis (sensu Eukarya)	6	2.00E-05
Factor 2 (674 genes)	Signal transduction	32	7.00E-05
	Immune response	30	0.0
	Cell adhesion	24	0.0
	Inflammatory response	17	0.0
	Chemotaxis	16	0.0
	Proteolysis and peptidolysis	15	0.002
	Cell growth and/or maintenance	13	0.002
	Cell-cell signaling	13	7.60E-04
	Cell proliferation	13	0.002
	Antimicrobial humoral response (sensu Vertebrata)	12	0.0
	G-protein coupled receptor protein signaling pathway	11	3.80E-04
	Cell motility	10	2.00E-05
	Cellular defense response	9	0.0
	Protein complex assembly	6	0.002
	Positive regulation of cell proliferation	6	0.008
	Cell-matrix adhesion	6	8.00E-05
	Blood coagulation	6	0.001
	Hetorophilic cell adhesion	6	0.002
Factor 3 (524 genes)	Signal transduction	22	0.004
	Protein folding	6	0.002
Factor 4 (610 genes)	Metabolism	16	1.00E-04
	Muscle development	11	1.00E-05
	Electron transport	10	0.005
	Carbohydrate metabolism	9	1.90E-04
	Muscle contraction	9	0.0
	DNA replication	6	0.002
	Energy pathways	6	4.20E-04
	Fatty acid metabolism	6	5.00E-05

Similarly, the fifth basis gene and basis experiment grouped together a set of samples related to blood and lymphoid tissues (for example samples from whole blood, different blood cell types, lymph node, tonsil, bone marrow or thymus) and a set of genes that were mainly involved in immune system related functions such as "inflammatory response", "cellular defense response" or "antigen representation, endogenous antigen". GO annotations related to signal transduction such as "signal transduction" or "intracellular signaling cascade" were also enriched in this gene module. Many of the genes annotated with these two last categories present in the module, such as *NCF4, LCP2, ITK, SYK, HA-1, TYROBP, TREM1 *and *STAT6*, are genes that play important roles in the immune system activation.

Finally, the last basis experiment defined a set of genes mainly involved in biological functions such as "spermatogenesis", "transcription" or "mitosis". These genes were over-expressed in five testis-related tissues (samples from testis interstitial, testis, testis seminiferous tubule, testis Leydig cells and testis germ cells) which were co-clustered by the corresponding basis gene. In addition, most of the probes annotated as testis specific genes included in the analysis, such as *PHKG2, TCTEL1, TEGT, TES, TSPY1 *or *TSPY2 *showed high coefficients in the eighth basis experiment, which supports the results obtained by our approach.

Therefore, the method was able to recover block-structures composed by subsets of genes that behave similarly in subsets of samples related to the same anatomic location or physiological function.

Additionally, our method performed better than standard hierarchical clustering analysis to uncover relationships among genes that share local similarities in their expression profiles with different sets of genes. For example, a set of genes that were over-expressed in testis tissues were clustered in distant positions to the testis-gene cluster by standard two-way clustering analysis due to global differences in their expression profiles (see additional file 1, figure 8). In contrast, our approach did succeed in grouping these genes in the testis-specific gene module (see figure 4c) or in very close positions.

Another example was provided by a set of 33 genes that were expressed in neural-and testis-derived tissues. Two-way hierarchical clustering grouped these genes in the same branch that the set of testis-specific genes, but their relationship with neural-expressed genes could not be inferred by the cluster dendrogram itself (see additional file 1, figures 8 and 9). These genes showed a peak in basis experiments 4 and 8 which, as we have shown before, were related to neural and testis tissues respectively. Our approach clustered these genes in close positions to both, the neural and testis gene modules (see additional file 1, figure 10). Among these genes were some that have been previously reported as testis and brain expressed genes such as *HSPA2 *[26] or *BSCL2*[27].

### Soft-tissue tumor dataset

One of the applications in which biclustering methods can provide interesting results is the analysis of cancer datasets. Local structures can be used to relate genes with specific tumor types or for classifying samples. We have analyzed the tumor dataset reported by Nielsen *et al*. [24], which comprises expression profiles of 46 samples corresponding to five different soft-tissue tumor types; 8 gastrointestinal stromal tumors, 8 monophasic synovial sarcomas, 4 liposarcomas, 11 leiomyosarcomas, 8 malignant fibrous histiocytomas and 2 benign peripheral nerve-sheath tumors (schwannomas). The aim of this analysis was to determine whether the proposed approach is able to recover the main block-structures associated to different tumor types.

We applied *ns*NMF to analyze this dataset with a rank value of 4, at which we could attest robustness of the model with a correspondingly high cophenetic coefficient. The four gene expression modules obtained at this rank showed a high consistency (all of them were found in more than 90% of the factorizations). The first basis gene grouped together the set of synovial sarcomas samples and the first basis experiment revealed the set of genes mainly over-expressed in this tumor cluster (figure 5a). Functional categories such as "regulation of transcription, DNA-dependent", "development" or "neurogenesis" were enriched in this set of genes (see table 1b). In the case of the last category, a related observation was reported by Nagayama *et al*., who analyzing the expression profile of different soft tissue tumor types also found that many over-expressed genes in synovial sarcomas were related to neural tissues and neural differentiation and they suggested a neuroectodermal origin of synovial sarcomas [28]. Additionally, many other genes that have been previously related to synovial sarcomas, such as the *SSX4 *gene, *EGFR *and *SALL2 *[29], components of the retinoic acid pathway (*CRABP1 *and *RARG*) as well as retinoic acid induced genes (*IRX5 *and *TGFβ2*) [24], were also included in this module.

The second basis gene clustered together a heterogeneous group of samples, including liposarcomas, some leiomyosarcomas and malignant fibrous histiocytosarcomas. An analysis of the corresponding basis experiment revealed a set of genes that were mainly annotated as "signal transduction" and with GO categories related to immune and defense response such as "immune response" or "inflammatory response". In addition, other biological processes such as "cell adhesion" or "cell-matrix adhesion" were also over-represented in this set of genes. These observations are highly consistent with the findings reported in the original paper, in which the authors found that a set of genes with fibrous and histiocytic features were related to this heterogeneous group of tumors. The histiocytic part included genes characteristically expressed by macrophages, genes of the interferon-responsive cluster and genes associated with other inflammatory processes. The fibrous part of the gene set included genes related to the extracellular matrix and angiogenesis such as many genes for collagen and collagen metabolism [24].

The third basis experiment and basis gene revealed a partition of 8 gastrointestinal stromal tumors and the genes that are relevant to induce this partition (figure 5c). GO categories of "signal transduction" and "protein folding" were over-represented in this set of genes. Among the genes involved in signal transduction the *KIT *gene, one of the main markers of gastrointestinal stromal tumors, showed a high coefficient in this basis experiment. Other markers of this type of tumors recently identified such as the gene *FLJ10261 *(*DOG1*) [30] or protein kinase C theta [31,32] also showed very high coefficients in the third basis experiment.

In a similar way, the last structure defined by the fourth basis gene and basis experiment revealed genes mainly over-expressed in six of the 11 leiomyosarcomas and one liposarcoma (figure 5d). We found that GO categories such as "metabolism, "muscle development" or "muscle contraction" were enriched in the set of genes belonging to this module. It is clear the relationship among the biological processes over-represented in this set of genes and the tissue origin of leiomyosarcomas samples.

These results demonstrate the ability of our algorithm to identify coherent substructures composed by sets of genes mainly expressed in samples related to the same tumor type. Furthermore, the proposed approach gave not only a partition of samples and genes based on these local patterns, but also an internal ranking of them within a given local structure revealing which genes are relevant to induce these partitions.

## Discussion

The standardization and extension of the use of microarray technology is allowing researches to generate larger and more heterogeneous gene expression datasets, some of them containing hundreds of different experimental conditions. The size and heterogeneity of these datasets has opened new challenges for the development of computational methods able to uncover local relationships among genes and conditions rather than patterns based on global similarities. This demand is supported, in part, by the fact that genes involved in the same biological process might only be co-expressed in a subset of experimental conditions and show uncorrelated expression levels in the rest of conditions.

In this paper we present an approach able to cluster genes and conditions that are highly related in sub-portions of the data. The method is based on the non negative matrix factorization technique, which has been previously applied to gene expression data analysis in a one-dimensional way [21,22]. Specifically, we have introduce a variant of the standard NMF algorithm, *ns*NMF [23], that is an attempt to improve the capacity of the classical NMF in the parts-based representation of the data by producing improved sparse components of the gene expression matrix.

We note that the local structures obtained by this approach correspond to sets of genes highly over-expressed in subsets of experimental conditions and the order vectors (columns of **W **and rows of **H**) indicate the relevance of genes and the experiments in each local pattern. Furthermore, genes and experimental conditions that show under-expression patterns or low expression values in these sub-parts of the data will show low coefficients in the corresponding basis gene and basis experiment respectively. Therefore, these genes and experiments will be placed in the upper right and bottom left corners of the array, although we can not assume that they are also sorted by their association to an under-expression pattern.

The results obtained in this study from the analysis of two large and heterogeneous gene expression datasets, as well as several synthetic datasets, illustrates the usefulness of our method. The approach was able to cluster genes and samples that showed a high consistency in their expression profiles in sub-portions of the data. Samples that were clustered together were related by identifiable criteria, such as samples derived from the same tumor type or physiologically related tissues. In the same way, the set of co-clustered genes were enriched in functional annotations that were in clear agreement with respect to the known biology about particular tissues or cellular types and provided insights into the main underlying biological process. Similar results were obtained from the analysis of other datasets (see the associated web site [25]) showing that this technique can be a useful tool to extract meaningful patterns from gene expression data.

In contrast to most of the currently available biclustering algorithms that apply greedy iterative searches to find significant patterns, imposing constraints on the size or the number of biclusters, our approach provides an alternative solution to uncover "natural" substructures that are related to the main patterns of the expression matrix. The use of matrix factorization methods in the context of biclustering is gaining attention by the bioinformatics community. Kluger *et al*. [33] suggested the use of singular value decomposition to find checkerboard patterns in gene expression matrices and more recently Dueck *et al*. [34] proposed a probabilistic sparse factorization method to discover gene expression biclusters. Both methods, although different in their nature, aims at extracting gene expression modules using matrix factorization techniques. The method proposed here can be included in this area, even though the objectives and constraints that it uses differ drastically from the ones proposed before. Similar to the situation that occurs when comparing different oneway clustering techniques, a fair comparison of biclustering algorithms is difficult to carry out due to the different criteria used for each technique that obviously leads to different but still valid results which might vary depending on the data and the criteria used by the methods. We conducted some empirical comparisons to illustrate this issue using other biclustering methods. Results can be found in the associated web site [25]. In this study we have shown that *ns*NMF can not only be successfully used in one dimensional way [21,22], but it can also be effectively applied to extract biological meaningful biclusters.

We consider important to mention that although some theoretical work on the properties of NMF models exist [35], much of the recent increasing interest of the method comes from its empirical success in extracting meaningful features from real data sets, where there is no guarantee that the data has a unique representation in terms of positive factors. This situation, as well as the non-deterministic nature of the method, is still an open problem that need a more extended work by the Pattern Recognition and Machine Learning community on both the theoretical and practical properties of the NMF family methods. Regardless of this problem, it is not an illegal practice to force-fit the NMF model to the data. Quite the contrary, the decomposition can be highly meaningful. The significant interpretation of the NMF model is the main reason that has motivated a recent explosion of applications in many fields, including for example gene expression [21,22], sequence analysis [36], functional characterization of gene lists [37] or text mining [38]. What this means is that, even if one does not know if the data at hand has a true, exact, unique, and recoverable non-negative factor structure, the model can be fitted to the data, and the extracted "parts" can be analyzed and interpreted for meaning. This is precisely the thrust of this work.

The number of extracted structures is directly related to the factorization rank. In this work we have estimated the factorization rank using the model selection method introduced by Brunet *et al*. [21], which is related to the cluster structure of samples. This estimation allows us to generate significant partitions of samples as well as identifying the set of genes that are important to induce these partitions. Nevertheless, higher ranks can reveal more localized patterns that might also be biologically relevant. In this sense, other estimations of the factorization rank such as the root-mean-square based estimation proposed by Kim and Tidor, [22] might also be interesting to be explored.

In this work we have also shown that the proposed approach was able to detect complex patterns and relationships among genes and conditions that were hidden when standard two-way clustering was used in the analysis of the expression matrix.

## Conclusion

In the present work we illustrate the application of the non-smooth non-negative matrix factorization technique for discovering local structures from gene expression datasets. Biclustering methods have centered the attention of many researchers in the field of gene expression data analysis due to their potentials to uncover meaningful relationships among genes and conditions. We hope this new method actively helps in the data analysis and knowledge discovery process in gene expression experiments.

## Methods

### Non negative matrix factorization

NMF is a matrix factorization algorithm originally introduced by Lee *et al*. to the analysis of facial images [39]. This technique can be applied to the analysis of multidimensional datasets in order to reduce the dimensionality, discover latent patterns and, more important, aid in the interpretation of the data.

The main difference between NMF and other classical factorization techniques that have been applied to gene expression data analysis, such as principal component analysis (PCA), singular value decomposition (SVD) or independent component analysis (ICA) relies in the non-negativity constraints imposed on both, the basis (**W**) and encoding vectors (**H**). These constraints tend to lead to a parts-based representation of the data because they allow only additive, not subtractive, combinations. In this way, factors can be interpreted as parts of the data or, in other words, as subsets of elements that tend to occur together in sub-portions of the dataset. On the contrary, other factorization techniques, like the ones mentioned above allow the entries of **W **and **H **to be of arbitrary sign, involving complex cancellations of positive and negative elements to reconstruct the original dataset. In other words, NMF tends to produce factors that lend themselves to a relatively easy contextual interpretation, while the factors obtained by the other mentioned approaches produce factors with no obvious contextual "meaning" in themselves.

Formally, the non-negative matrix decomposition can be described as follow:

**V ≈ WH **Eq. 1

where **V **∈ ℝ^*m*×*n *^is a positive data matrix with *m *variables and *n *objects, **W **∈ ℝ^*m*×*k *^are the reduced *k *basis vectors or factors, and **H **∈ ℝ^*k*×*n *^contains the coefficients of the linear combinations of the basis vectors needed to reconstruct the original data (also known as encoding vectors). Additionally we have the following conditions: *k *≤ *m*, all matrices **V**, **W**, **H **are non-negative, and the columns of **W **(the basis vectors) are normalized (sum up to 1). As we have mentioned, the main difference between NMF and other classical factorization models relies in the non-negativity constraints imposed on both the basis **W **and encoding vectors **H**. In this way, only additive combinations are possible:

(V)iμ≈(WH)iμ=∑a=1kWiaHaμ     Eq. 2
 MathType@MTEF@5@5@+=feaafiart1ev1aaatCvAUfKttLearuWrP9MDH5MBPbIqV92AaeXatLxBI9gBaebbnrfifHhDYfgasaacH8akY=wiFfYdH8Gipec8Eeeu0xXdbba9frFj0=OqFfea0dXdd9vqai=hGuQ8kuc9pgc9s8qqaq=dirpe0xb9q8qiLsFr0=vr0=vr0dc8meaabaqaciaacaGaaeqabaqabeGadaaakeaacqGGOaakieqacqWFwbGvcqGGPaqkdaWgaaWcbaGaemyAaKgcciGae4hVd0gabeaakiabgIKi7kabcIcaOiab=Dfaxjab=HeaijabcMcaPmaaBaaaleaacqWGPbqAcqGF8oqBaeqaaOGaeyypa0ZaaabCaeaacqWGxbWvdaWgaaWcbaGaemyAaKMaemyyaegabeaakiabdIeainaaBaaaleaacqWGHbqycqGF8oqBaeqaaaqaaiabdggaHjabg2da9iabigdaXaqaaiabdUgaRbqdcqGHris5aOGaaCzcaiaaxMaacqqGfbqrcqqGXbqCcqqGUaGlcqqGGaaicqqGYaGmaaa@525F@

The objective function, based on the Poisson likelihood, can be defined using the following divergence function, which we need to minimize:

D(V,WH)=∑i=1m∑j=1n(Vijln⁡Vij(WH)ij−Vij+(WH)ij)     Eq. 3
 MathType@MTEF@5@5@+=feaafiart1ev1aaatCvAUfKttLearuWrP9MDH5MBPbIqV92AaeXatLxBI9gBaebbnrfifHhDYfgasaacH8akY=wiFfYdH8Gipec8Eeeu0xXdbba9frFj0=OqFfea0dXdd9vqai=hGuQ8kuc9pgc9s8qqaq=dirpe0xb9q8qiLsFr0=vr0=vr0dc8meaabaqaciaacaGaaeqabaqabeGadaaakeaacqWGebarcqGGOaakieqacqWFwbGvcqGGSaalcqWFxbWvcqWFibascqGGPaqkcqGH9aqpdaaeWbqaamaaqahabaWaaeWaaeaacqWGwbGvdaWgaaWcbaGaemyAaKMaemOAaOgabeaakiGbcYgaSjabc6gaUnaalaaabaGaemOvay1aaSbaaSqaaiabdMgaPjabdQgaQbqabaaakeaacqGGOaakcqWFxbWvcqWFibascqGGPaqkdaWgaaWcbaGaemyAaKMaemOAaOgabeaaaaGccqGHsislcqWGwbGvdaWgaaWcbaGaemyAaKMaemOAaOgabeaakiabgUcaRiabcIcaOiab=Dfaxjab=HeaijabcMcaPmaaBaaaleaacqWGPbqAcqWGQbGAaeqaaaGccaGLOaGaayzkaaaaleaacqWGQbGAcqGH9aqpcqaIXaqmaeaacqWGUbGBa0GaeyyeIuoakiaaxMaacaWLjaGaeeyrauKaeeyCaeNaeeOla4IaeeiiaaIaee4mamdaleaacqWGPbqAcqGH9aqpcqaIXaqmaeaacqWGTbqBa0GaeyyeIuoaaaa@698E@

Solving the problem described in the previous equation, the derived algorithm is as follows:

1. Initialize **W **and **H **with positive random numbers.

2. For each basis vector **W**_*a *_∈ ℝ^*mx*1^, update the corresponding encoding vector **H**_*a *_∈ ℝ^1*xn*^; followed by updating and normalizing the basis vector **W**_*a*_. Repeat this process until convergence.

Iteration of the rules described above converges to a local minimum of the objective function described in equation 3. Formally, the detailed algorithm follows:

Repeat until convergence:

For a = 1 ...*k *do begin

For b = 1 ...*n *do

Hab←Hab∑i=1m(WiaVib)/∑q=1kWiqHqb∑i=1mWia     Eq. 4
 MathType@MTEF@5@5@+=feaafiart1ev1aaatCvAUfKttLearuWrP9MDH5MBPbIqV92AaeXatLxBI9gBaebbnrfifHhDYfgasaacH8akY=wiFfYdH8Gipec8Eeeu0xXdbba9frFj0=OqFfea0dXdd9vqai=hGuQ8kuc9pgc9s8qqaq=dirpe0xb9q8qiLsFr0=vr0=vr0dc8meaabaqaciaacaGaaeqabaqabeGadaaakeaacqWGibasdaWgaaWcbaGaemyyaeMaemOyaigabeaakiabgcziSkabdIeainaaBaaaleaacqWGHbqycqWGIbGyaeqaaOWaaSaaaeaadaaeWbqaamaalyaabaWaaeWaaeaacqWGxbWvdaWgaaWcbaGaemyAaKMaemyyaegabeaakiabdAfawnaaBaaaleaacqWGPbqAcqWGIbGyaeqaaaGccaGLOaGaayzkaaaabaWaaabCaeaacqWGxbWvdaWgaaWcbaGaemyAaKMaemyCaehabeaakiabdIeainaaBaaaleaacqWGXbqCcqWGIbGyaeqaaaqaaiabdghaXjabg2da9iabigdaXaqaaiabdUgaRbqdcqGHris5aaaaaSqaaiabdMgaPjabg2da9iabigdaXaqaaiabd2gaTbqdcqGHris5aaGcbaWaaabCaeaacqWGxbWvdaWgaaWcbaGaemyAaKMaemyyaegabeaaaeaacqWGPbqAcqGH9aqpcqaIXaqmaeaacqWGTbqBa0GaeyyeIuoaaaGccaWLjaGaaCzcaiabbweafjabbghaXjabb6caUiabbccaGiabbsda0aaa@67B4@

For c = 1 ...*m *do begin

Wca←Wca∑j=1m(HajVcj)/∑q=1kWcqHqj∑j=1nHaj     Eq. 5
 MathType@MTEF@5@5@+=feaafiart1ev1aaatCvAUfKttLearuWrP9MDH5MBPbIqV92AaeXatLxBI9gBaebbnrfifHhDYfgasaacH8akY=wiFfYdH8Gipec8Eeeu0xXdbba9frFj0=OqFfea0dXdd9vqai=hGuQ8kuc9pgc9s8qqaq=dirpe0xb9q8qiLsFr0=vr0=vr0dc8meaabaqaciaacaGaaeqabaqabeGadaaakeaacqWGxbWvdaWgaaWcbaGaem4yamMaemyyaegabeaakiabgcziSkabdEfaxnaaBaaaleaacqWGJbWycqWGHbqyaeqaaOWaaSaaaeaadaaeWbqaamaalyaabaWaaeWaaeaacqWGibasdaWgaaWcbaGaemyyaeMaemOAaOgabeaakiabdAfawnaaBaaaleaacqWGJbWycqWGQbGAaeqaaaGccaGLOaGaayzkaaaabaWaaabCaeaacqWGxbWvdaWgaaWcbaGaem4yamMaemyCaehabeaakiabdIeainaaBaaaleaacqWGXbqCcqWGQbGAaeqaaaqaaiabdghaXjabg2da9iabigdaXaqaaiabdUgaRbqdcqGHris5aaaaaSqaaiabdQgaQjabg2da9iabigdaXaqaaiabd2gaTbqdcqGHris5aaGcbaWaaabCaeaacqWGibasdaWgaaWcbaGaemyyaeMaemOAaOgabeaaaeaacqWGQbGAcqGH9aqpcqaIXaqmaeaacqWGUbGBa0GaeyyeIuoaaaGccaWLjaGaaCzcaiabbweafjabbghaXjabb6caUiabbccaGiabbwda1aaa@67CC@

Wca←Wca∑j=1nWja     Eq. 6
 MathType@MTEF@5@5@+=feaafiart1ev1aaatCvAUfKttLearuWrP9MDH5MBPbIqV92AaeXatLxBI9gBaebbnrfifHhDYfgasaacH8akY=wiFfYdH8Gipec8Eeeu0xXdbba9frFj0=OqFfea0dXdd9vqai=hGuQ8kuc9pgc9s8qqaq=dirpe0xb9q8qiLsFr0=vr0=vr0dc8meaabaqaciaacaGaaeqabaqabeGadaaakeaacqWGxbWvdaWgaaWcbaGaem4yamMaemyyaegabeaakiabgcziSoaalaaabaGaem4vaC1aaSbaaSqaaiabdogaJjabdggaHbqabaaakeaadaaeWbqaaiabdEfaxnaaBaaaleaacqWGQbGAcqWGHbqyaeqaaaqaaiabdQgaQjabg2da9iabigdaXaqaaiabd6gaUbqdcqGHris5aaaakiaaxMaacaWLjaGaeeyrauKaeeyCaeNaeeOla4IaeeiiaaIaeeOnaydaaa@4812@

End

End

### Non-smooth Non Negative Matrix Factorization (*ns*NMF)

Even if NMF has been presented and used as a method capable of finding the underlying component-based structure of complex data, there is no explicit guarantee in the method to support this property, other than the non-negativity constraints. In fact, taking a closer look at the basis and encoding vectors produced by the original NMF model [39], it is noticeable that there is a high degree of overlapping among basis vectors that contradict the intuitive nature of the "parts" [40]. As a consequence, a further evolution of NMF capable of producing more localized feature representations of both genes and experiments is highly desirable in this type of application.

In this direction, there are several reported attempts for solving this problem by making modifications to the original NMF functional to enforce sparseness on the basis vectors, the encoding vectors, or both [41-43].

In this work we decided to use a recent sparse non-negative factorization technique whose cost function is derived by introducing a modification to the original NMF model (equation 1) in order to demand sparseness to both, the basis and encoding vectors. The new method, here referred to as Non-smooth Non-Negative Matrix Factorization (*ns*NMF) [23], differs from the original in the use of an extra smoothness matrix to impose sparseness. A full comparison of this method with the other reported sparse versions of NMF [41-44] can be found in [23] whose results reflects the superiority of *ns*NMF in finding sparse factors without drastically affecting the quality of the factorization process.

The goal of *ns*NMF is to find sparse structures in the basis functions that better explain the data set. The interpretation of the new factorization is then two fold: data can be faithful reconstructed using additive combinations of a reduced set of factors and, at the same time, interpretation of the factors is easier due to the intuitive sparse, non-overlapped part-based representation of the data.

In order to get sparseness, the *ns*NMF model demands a smooth distribution of the factors. This is achieved by changing the model of equation 1 into:

**V **≈ **WSH **Eq. 7

where **S **∈ ℝ^*k*×*k *^is a positive smoothness matrix defined as:

S=(1−ϑ)I+θ11tk     Eq. 8
 MathType@MTEF@5@5@+=feaafiart1ev1aaatCvAUfKttLearuWrP9MDH5MBPbIqV92AaeXatLxBI9gBaebbnrfifHhDYfgasaacH8akY=wiFfYdH8Gipec8Eeeu0xXdbba9frFj0=OqFfea0dXdd9vqai=hGuQ8kuc9pgc9s8qqaq=dirpe0xb9q8qiLsFr0=vr0=vr0dc8meaabaqaciaacaGaaeqabaqabeGadaaakeaaieqacqWFtbWucqGH9aqpcqGGOaakcqaIXaqmcqGHsisliiGacqGFrpGscqGGPaqkcqWFjbqscqGHRaWkcqGF4oqCdaWcaaqaaiab=fdaXiab=fdaXmaaCaaaleqabaGaemiDaqhaaaGcbaGaem4AaSgaaiaaxMaacaWLjaGaeeyrauKaeeyCaeNaeeOla4IaeeiiaaIaeeioaGdaaa@4311@

where **I **is the identity matrix, **1 **∈ ℝ^1×*k *^is a column vector of 1s, the superscript ^t ^indicates vector transpose and the parameter *θ *controls the sparseness of the model, satisfying 0 ≤ *θ *≤ 1.

Finally the new objective function for the *ns*NMF model can be formally described as:

D(V,WSH)=∑i=1m∑j=1n(Vijln⁡Vij(WSH)ij−Vij+(WSH)ij)     Eq. 9
 MathType@MTEF@5@5@+=feaafiart1ev1aaatCvAUfKttLearuWrP9MDH5MBPbIqV92AaeXatLxBI9gBaebbnrfifHhDYfgasaacH8akY=wiFfYdH8Gipec8Eeeu0xXdbba9frFj0=OqFfea0dXdd9vqai=hGuQ8kuc9pgc9s8qqaq=dirpe0xb9q8qiLsFr0=vr0=vr0dc8meaabaqaciaacaGaaeqabaqabeGadaaakeaacqWGebarcqGGOaakieqacqWFwbGvcqGGSaalcqWFxbWvcqWFtbWucqWFibascqGGPaqkcqGH9aqpdaaeWbqaamaaqahabaWaaeWaaeaacqWGwbGvdaWgaaWcbaGaemyAaKMaemOAaOgabeaakiGbcYgaSjabc6gaUnaalaaabaGaemOvay1aaSbaaSqaaiabdMgaPjabdQgaQbqabaaakeaacqGGOaakcqWFxbWvcqWFtbWucqWFibascqGGPaqkdaWgaaWcbaGaemyAaKMaemOAaOgabeaaaaGccqGHsislcqWGwbGvdaWgaaWcbaGaemyAaKMaemOAaOgabeaakiabgUcaRiabcIcaOiab=Dfaxjab=nfatjab=HeaijabcMcaPmaaBaaaleaacqWGPbqAcqWGQbGAaeqaaaGccaGLOaGaayzkaaGaaCzcaiaaxMaacqqGfbqrcqqGXbqCcqqGUaGlcqqGGaaicqqG5aqoaSqaaiabdQgaQjabg2da9iabigdaXaqaaiabd6gaUbqdcqGHris5aaWcbaGaemyAaKMaeyypa0JaeGymaedabaGaemyBa0ganiabggHiLdaaaa@6D11@

The interpretation of **S **as a smoothing matrix can be explained as follows. Let **X **be a positive, non-zero, vector. Consider the transformed vector **Y **= **SX**. If *θ *= 0, then **Y **= **X**, and no smoothing on **X **has occurred. However, as *θ *→ 1, the vector **Y **tends to the constant vector with all elements almost equal to the average of the elements of **X**. This is the smoothest possible vector, in the sense of "non-sparseness", because all entries are equal to the same non-zero value, instead of having some values close to zero and others clearly non-zero. Note that when *θ *= 0, the model corresponds to the basic NMF.

Further insight into the nature of the new *ns*NMF model can be obtained from the dual interpretation of Eq. 7, which can be equivalently written as:

**V **= **(WS)H **= **W(SH)**

Non-sparseness in the basis **W **will force sparseness in the encoding **H**. At the same time, non-sparseness in the encoding **H **will force sparseness in the basis **W**. Due precisely to the simultaneity of both conditions, sparseness will be enforced on both basis and encoding parts. This property of *ns*NMF is the main motivation for using this algorithm for biclustering, due to its ability in extracting local (sparse) patterns from the data.

The new algorithm is very straightforward to derive by taking partial derivatives of the functional in equation 9 with respect to **H **and **W **and setting them to zero respectively [23]. As it was expected, for a given sparseness parameter value 0 ≤ *θ *≤ 1, the final algorithm is a simple modification of the original, basic NMF algorithm given by Eqs. 4–6:

1. In Eq. 4 (update for **H**), substitute **W **by **WS**.

2. In Eq. 5 (update for **W**), substitute **H **by **SH**.

Intuitively the algorithm obtained after minimizing the functional in equation 9 reflects the nature of the *ns*NMF: to estimate the encoding vectors **H**, the algorithm takes into account the smooth version of **W **(given by **WS**). In a similar manner, to estimate the values of the basis vectors **W**, the algorithm used the smooth version of **H **(given by **SH**). When these two conditions are used simultaneously, sparse versions of **W **and **H **are obtained.

It is important to mention that the parameter *θ *controls the sparseness of the model and cannot be estimated from the functional, in the sense that it is a hyper-parameter. In this work we have performed numerous empirical tests, and found that the value of 0.5 leads to reasonable results without affecting very much the explained variance of the model.

### Selection of the factorization rank

An important consideration in the application of *ns*NMF, and also in the classical NMF model, is the selection of the number of factors needed to better represent the data. Generally, as a rule of thumb, this value is generally chosen so that (*n + m*) *k *<*nm *and thus the product **WH **can be regarded as a compressed form of the data in **V **[39]. Nevertheless, this estimation is not informative enough to make a proper decision. Finding an appropriate value of *k *depends on the application and it is mostly influenced by the nature of the dataset itself. It is intuitively evident that the more factors we use, the more detailed information we get. However, since the main goal of this application is to automatically extract a number significant block-structures related to the main biological patterns in the dataset, it is important to use only a reduced set of factors that explain the data enough without obscuring the biclusters information with too many details.

In addition, another important aspect to take into account when selecting the number of factors in *ns*NMF is that the sparseness of the model is less evident if only a few factors are used. This is a natural consequence of any factorization method: the fewer factors we use, the most informative the factors should be in order to explain, in the best possible way, the original data

In this work, we used the model selection method proposed by Brunet *et al*. [21] to estimate the value of *k*. They used the cophenetic correlation coefficient as a measure of the stability of the model for different values of *k *with respect to different random initial conditions. The values of *k *where the cophenetic coefficient shows the highest value or begins to fall reflects stability in the results with respect to the differences in the initial conditions [21].

### Gene selection in basis experiments

Sorting the matrix by basis genes and basis experiments creates a natural ordination in which genes and samples are arranged based on their association to a given local pattern. The challenge now is to determine the partition corresponding to the set of genes and experiments that best define the local feature captured by the algorithm. One of the advantages of the factorization model we are using is that the sparse nature the *ns*NMF algorithm reinforces those genes and experiments that significantly sustain the factor while masks those that do not add any value to it at the same time. However if a small number of factors are used, the *ns*NMF algorithm will try to explain the data in the best possible way, consequently producing a large set of genes and conditions in the resulting biclusters. This situation is especially evident in the case of genes due to the high dimensional gene space of this type of matrices. Therefore, an additionally selection of the most representative genes, out of the sorted list produced by the algorithm, is still needed. In related works some authors have applied different criteria to select the most representative genes in each factor. For example we can impose a threshold in gene coefficients to obtain a reduced number of genes in each factor [22] or simply select a determined number of genes in each factor [45]. In this work we defined as factor-specific genes those genes that show high coefficients for a given factor and at the same time they show low coefficients for the rest. Operationally, this was achieved by sorting the genes in descending order by their coefficients in a given column of **W **(column *j*) and selecting only the first consecutive genes from the sorted list whose highest entry in **W **was the coefficient in column *j*. This procedure was repeated for each column of **W **and the set of genes selected in each case define a gene expression module.

### Consistency of gene modules

The set of genes contained in each module can show variations across different runs of the algorithm because of differences in the initial conditions. Furthermore, although the rank used in the factorization is highly related to the cluster structure of conditions, some groups of conditions may not be characterized by a well defined gene expression signature and the set of genes belonging to the corresponding module can vary among different runs of the algorithm. We can assume that a gene module is consistent, and represent a coherent structure, when it can be recovered independently of the initial random conditions. To identify consistent modules we run the algorithm 100 times and selected the set of results obtained in the factorization leading to the largest explained variance for further analysis. We then evaluated the repetition of the *k *gene modules obtained in this factorization across the remaining 99 factorizations. This was achieved by comparing the genes contained in each module. We consider that two modules were similar and were repeated when they shared 75% of their genes (90% in the case of synthetic data). A module was then considered consistent when it was found in more than 80% of factorizations.

### Datasets and data preprocessing

#### Simulated data

Synthetic data containing different overlapping and non-overlapping structures were generated to assess the performance of our method. Homogeneous block-structures were generated by random numbers with N(3,1) distribution. Background noise consisted in a 100 × 20 random matrix with N(0,1) distribution. The first dataset (A) contains two non-overlapped patterns of size 20 × 5 and 25 × 8 respectively. The second dataset (B) contains three overlapped patterns of size 10 × 8, 15 × 9 and 20 × 5 respectively and one non-overlapped pattern of size 10 × 3.

#### Human tissue dataset

Su *et al*. used human Affymetrix high-density oligonucleotide arrays to determine the gene expression profiles of 79 different human tissue samples and cell lines [6]. Data containing gene expression levels monitored with the Human U133 Affymetrix GeneChip across human tissues were obtained from the Gene Expression Omnibus repository [46]. The samples and replicates representing the same tissue or cell line were averaged. To exclude genes with minimal variation across samples, we removed those probe sets that did not satisfy the max-min < 1000 and max/min > 10 thresholds (max and min represent the maximum and minimum expression values for each gene respectively). Additionally, we also eliminated those genes that did not show an expression value greater than or equal to 1000 in at least 5 conditions.

#### Soft-tissue tumor dataset

This dataset was generated by Nielsen *et al*. [24] and contains expression profiles of different soft-tissue tumor types, including 8 gastrointestinal stromal tumors, 8 monophasic synovial sarcomas, 4 liposarcomas, 11 leiomyosarcomas, 8 malignant fibrous histiocytomas and 2 benign peripheral nerve-sheath tumors (schwannomas). They analyzed a gene expression dataset that contains expression levels of 5520 well defined genes across 46 samples (five tumor samples were hybridized in duplicate). We applied our method to the analysis of this tumor dataset which is available at [47]. Genes with absent values in more than 6 samples were filtered out and the remaining missing values were filled out using the *k*-nearest neighborhood approach (*k *= 10) [48].

After pre-processing, each gene expression dataset was normalized as Getz *et al*. described [16] and exponentially scaled to fit the positive constraints of the model.

### Analysis of biological annotation enrichment

To evaluate the enrichment of functional annotations in the set of genes contained in each module we used the Onto-Express tool [49]. This tool uses the hypergeometric test to estimate the statistical significance of the enrichment of a given GO term in a list of genes with respect to a reference list. We used the "biological process" category of the GO ontology to assign a biological meaning to each module. As a reference list we used the full set of genes in the array. Categories with *p-*values less than 0.01 were considered statistically significant.

## Authors' contributions

PCS and APM conceived the study. PCS carried out the computational analysis. RDPM and APM designed and developed the *ns*NMF algorithm. FT developed the computational optimization of the method. JMC and APM managed and coordinated the project. All authors participated in writing and revising the final manuscript.

## Supplementary Material

Additional File 1 A PDF file containing additional figures mentioned in the main manuscript Click here for file
