# Supplementary material for: Biclustering of gene expression data by non-smooth non-negative matrix factorization
Source: BMC Bioinformatics. 2006 Feb 17;7:78. doi: 10.1186/1471-2105-7-78 (PMC1434777; doi:10.1186/1471-2105-7-78)
Supplement: Additional File 1 — A PDF file containing additional figures mentioned in the main manuscript [file 1471-2105-7-78-S1.pdf]

ADDITIONAL FIGURES

a **b**

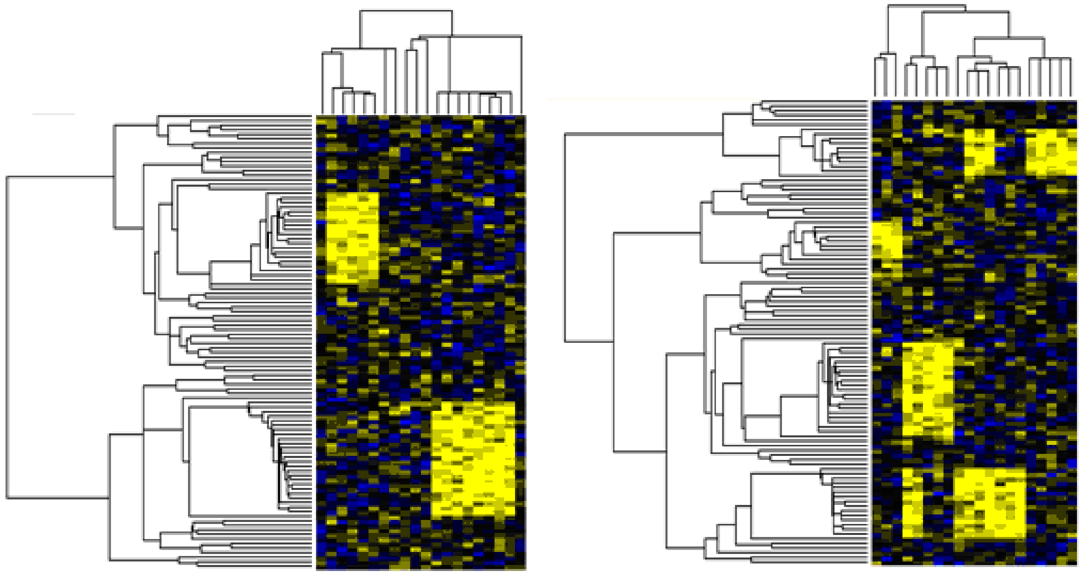

Additional figure 1. Two-way average linkage hierarchical clustering of (a) synthetic dataset A and (b) synthetic dataset B. Pearson correlation was used as similarity measure.

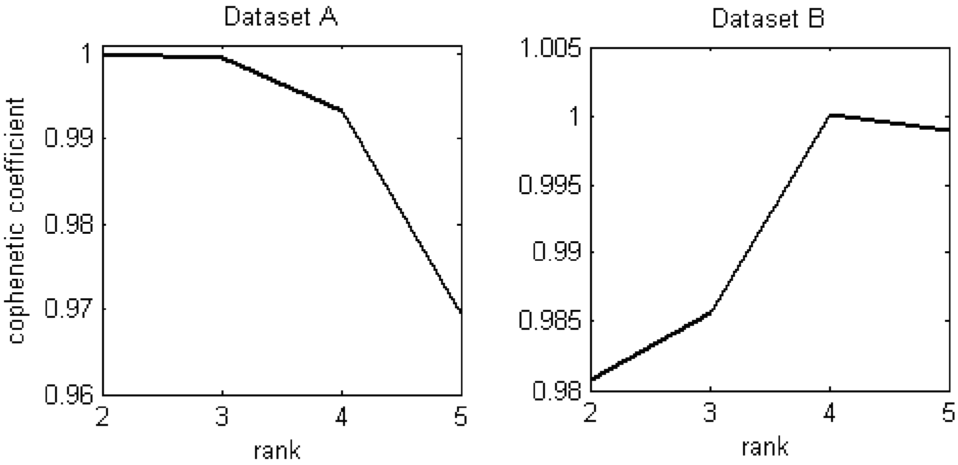

Additional figure 2. Cophenetic correlation coefficient computed at different ranks for synthetic datasets A and B.

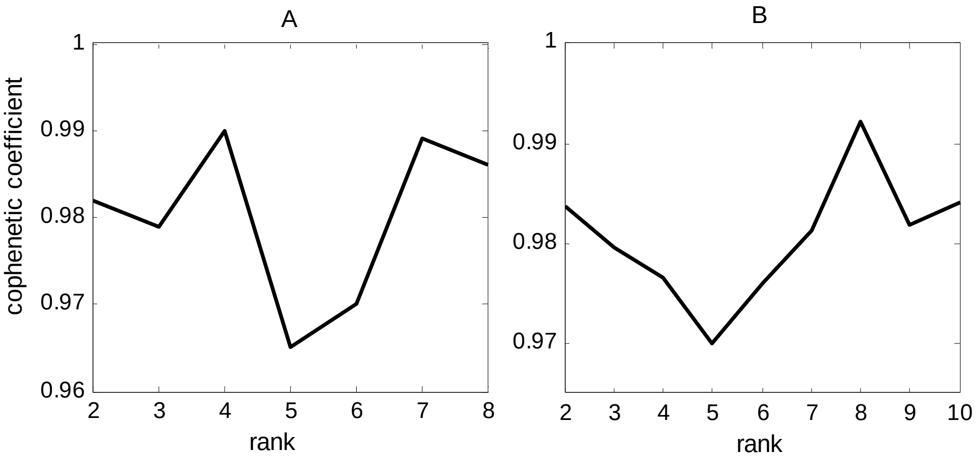

Additional figure 3. Cophenetic correlation coefficient computed at different ranks for (A) the soft tissue tumor dataset and (B) the human transcriptome dataset.

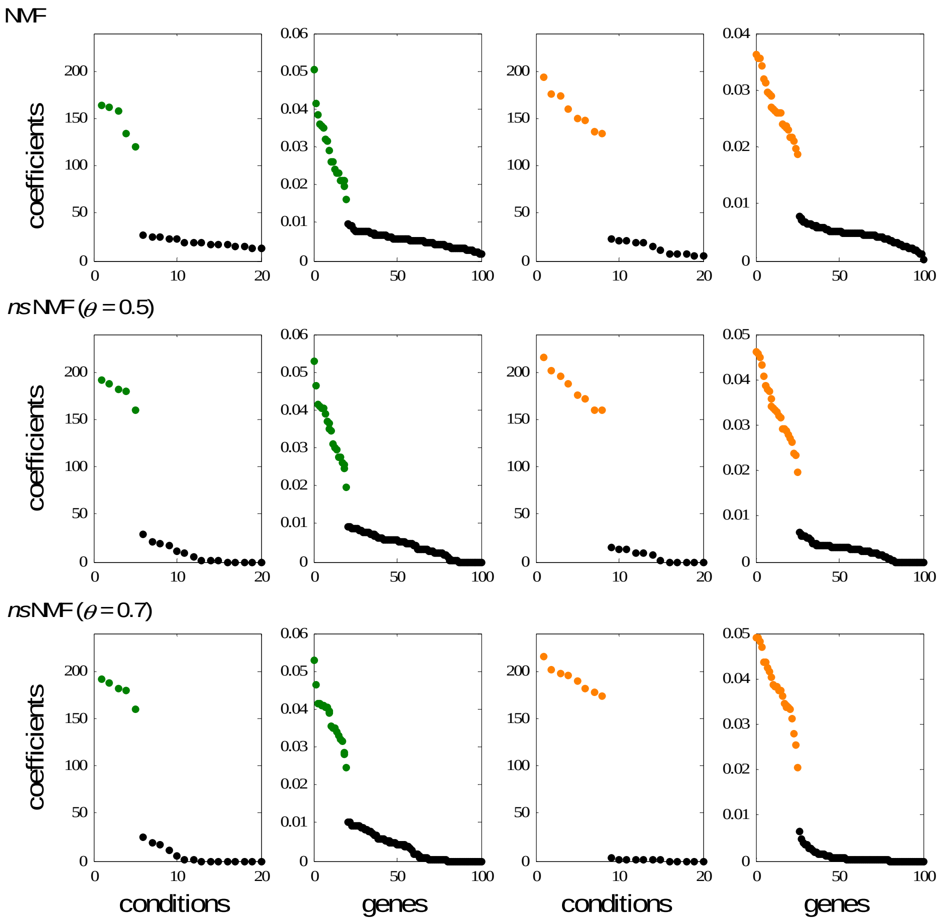

Additional figure 4. Comparative analysis of NMF and *ns*NMF (with sparseness parameter θ set to 0.5 and 0.7) algorithms applied to the synthetic dataset A with k=3. Only the two basis genes and basis experiments that captured the patterns P1a and P2a are shown. Plots in the first and third columns correspond to conditions arranged by decreasing entries in basis genes (rows of H). Plots in the second and fourth columns correspond to genes arranged by decreasing entries in basis experiments (columns of W). Plots in the first two columns correspond to basis genes and basis experiments that recovered P1a and the third and fourth columns correspond to those that recovered P2a. Conditions and genes belonging to P1a are marked in green and conditions and genes belonging to P1b are marked in orange.

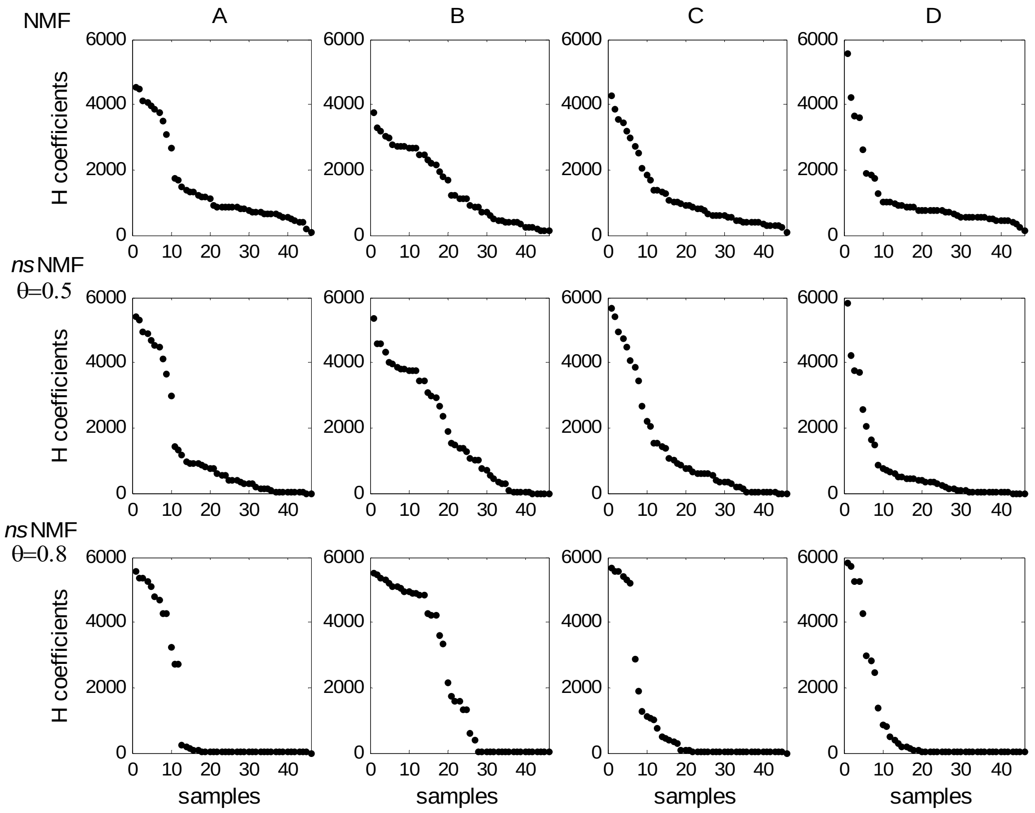

Additional figure 5. Comparative analysis of NMF and *ns*NMF algorithms applied to the soft-tissue tumor dataset with k=4. Plots correspond to conditions arranged by decreasing entries in basis genes (rows of **H**). A: basis gene corresponding to gastrointestinal stromal samples, B: basis gene corresponding to the heterogeneous tumor set, C: basis gene corresponding to monophasic synovial sarcomas D: basis gene corresponding to leiomyosarcomas samples.

A

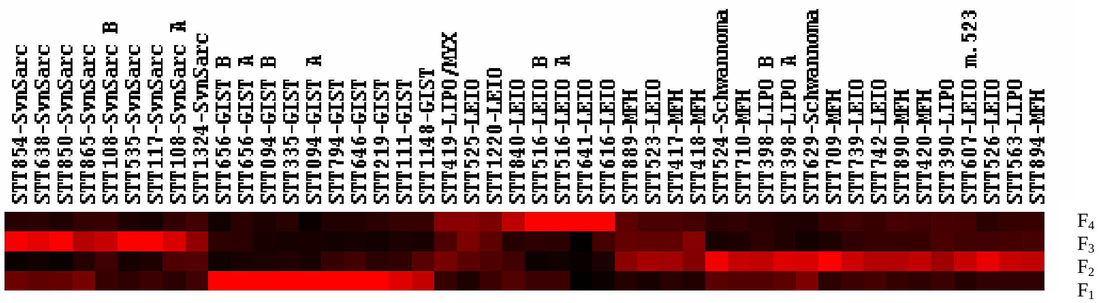

B

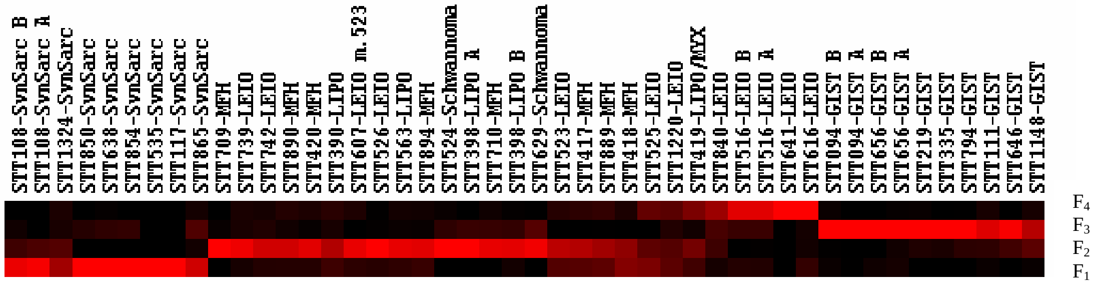

C

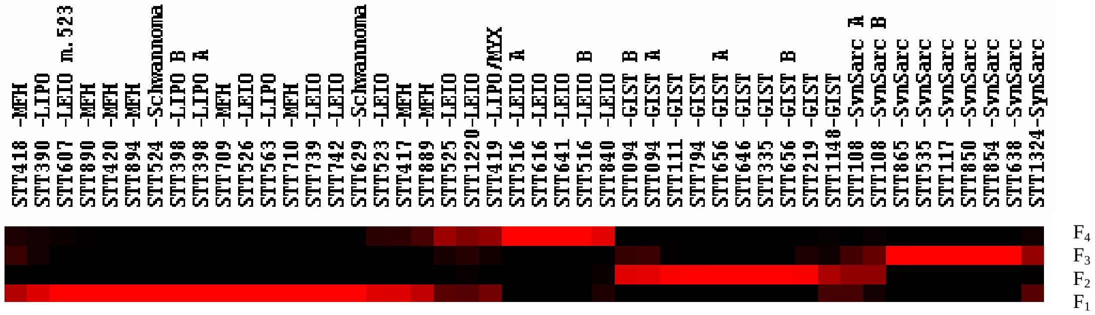

Additional figure 6. Hierarchical clustering of soft-tissue tumor samples based on their coefficients in basis genes (rows of **H**) yielded by standard NMF (A), *ns*NMF with a sparseness constant (θ) equal to 0.5 (B) and *ns*NMF with a sparseness constant (θ) equal to 0.8. The color scale ranges from 0 (black color) to the maximum value in each matrix (dark red). Note that while encodings yielded by standard NMF are not really sparse *ns*NMF produces more compact representations of the original experiments. That is, NMF relatively needs to linearly combine different factors to represent an original experiment while *ns*NMF tends to explain the same experiment by the additive combination of just a few clearly independent gene expression features.

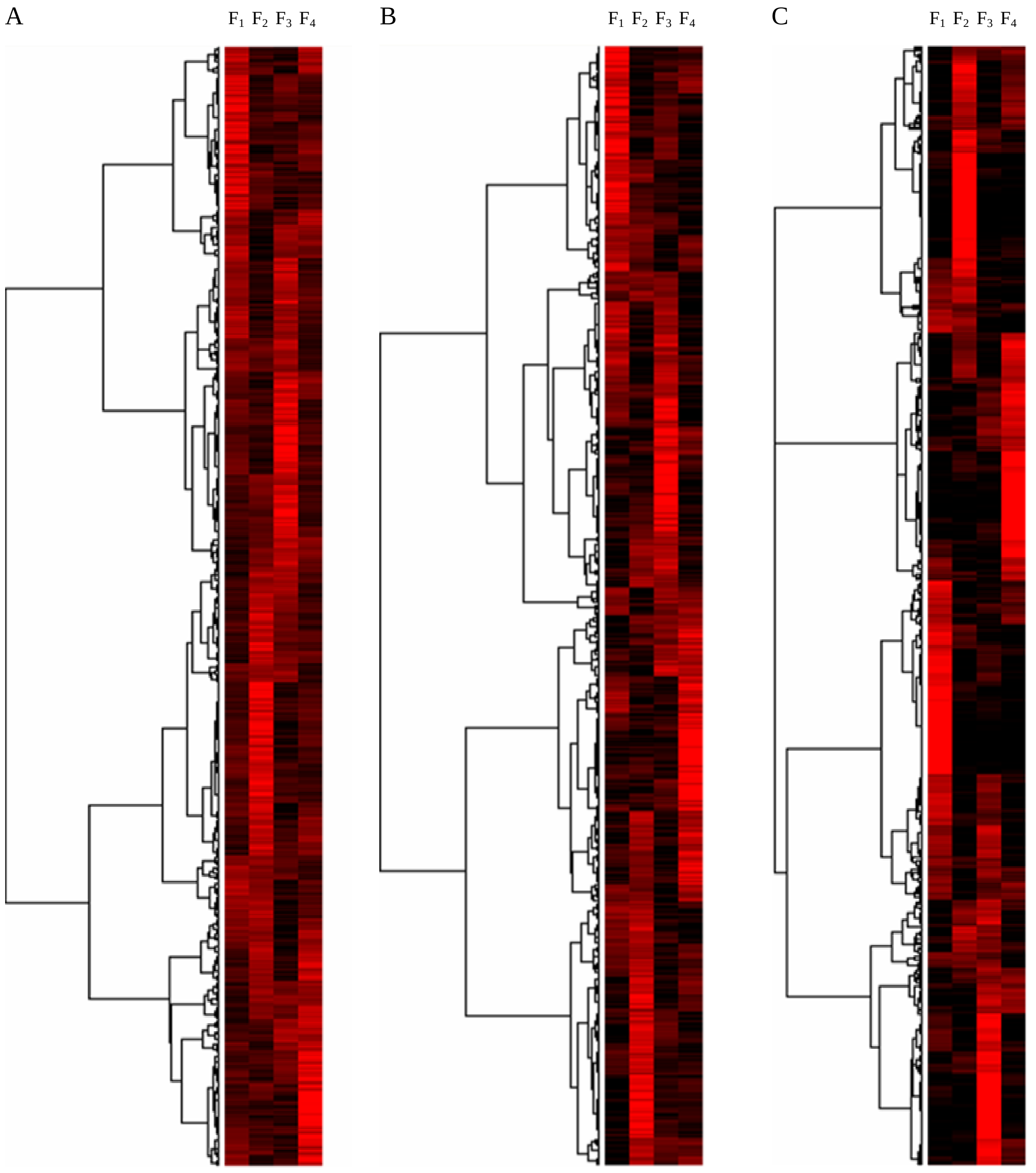

Additional figure 7. Hierarchical clustering of genes based on their coefficients in basis experiment (columns of **W**) yielded by matrix factorization by standard NMF (A), *ns*NMF with θ=0.5 (B) and *ns*NMF with θ=0.8. As in the above case, *ns*NMF produces a more evident sparse representation of basis experiments (factors) allowing us to get clearer sets of genes belonging to the same gene expression feature.

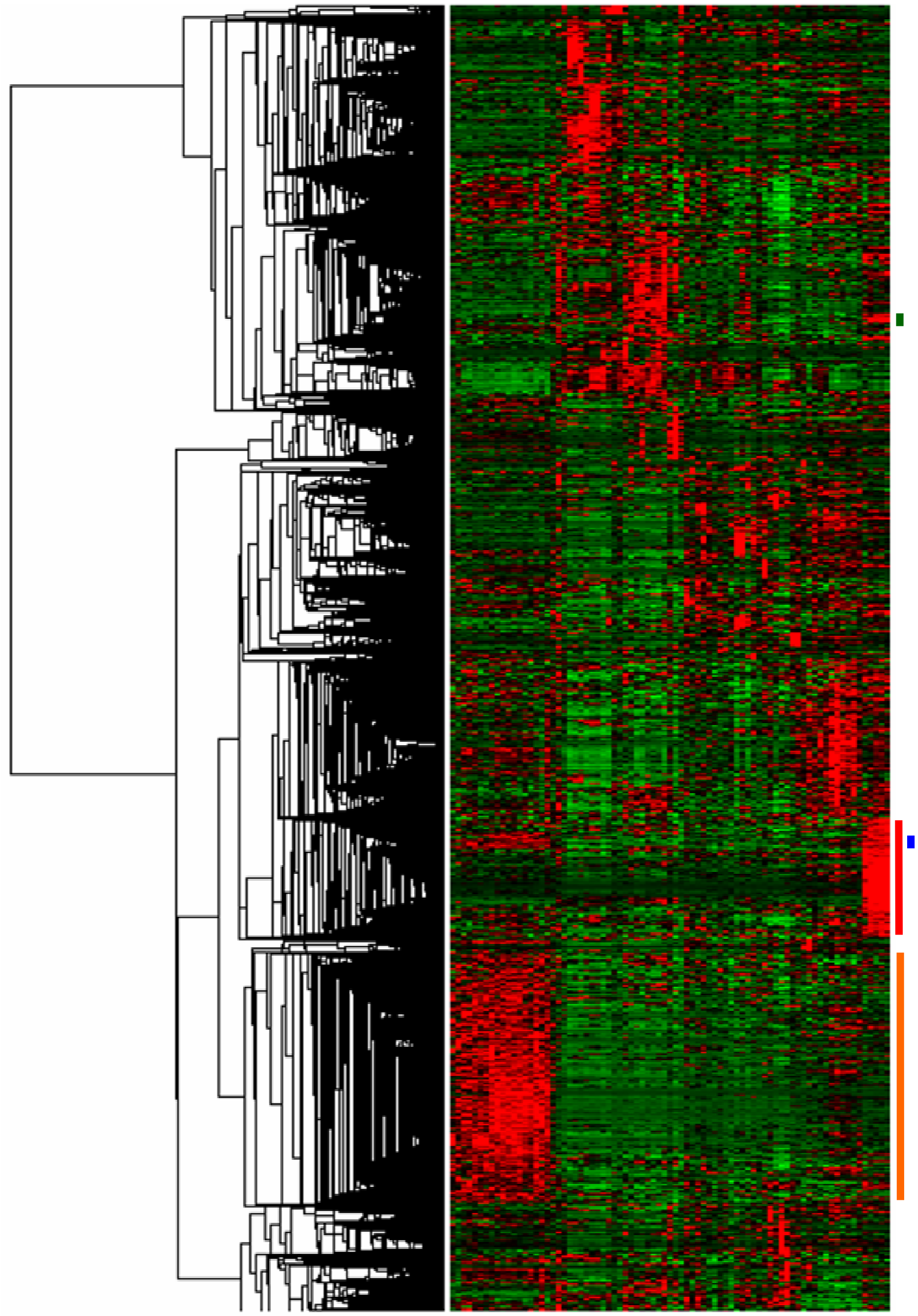

Additional figure 8. Two-way hierarchical clustering of human transcriptome dataset. Genes that are expressed in neural tissues are marked in orange, genes that are expressed in testis-derived tissues are marked in red and genes that are expressed in both tissue clusters are marked in blue. A set of genes that are expressed in testis tissues but, are clustered in a distant position to the testis gene cluster, are marked green.

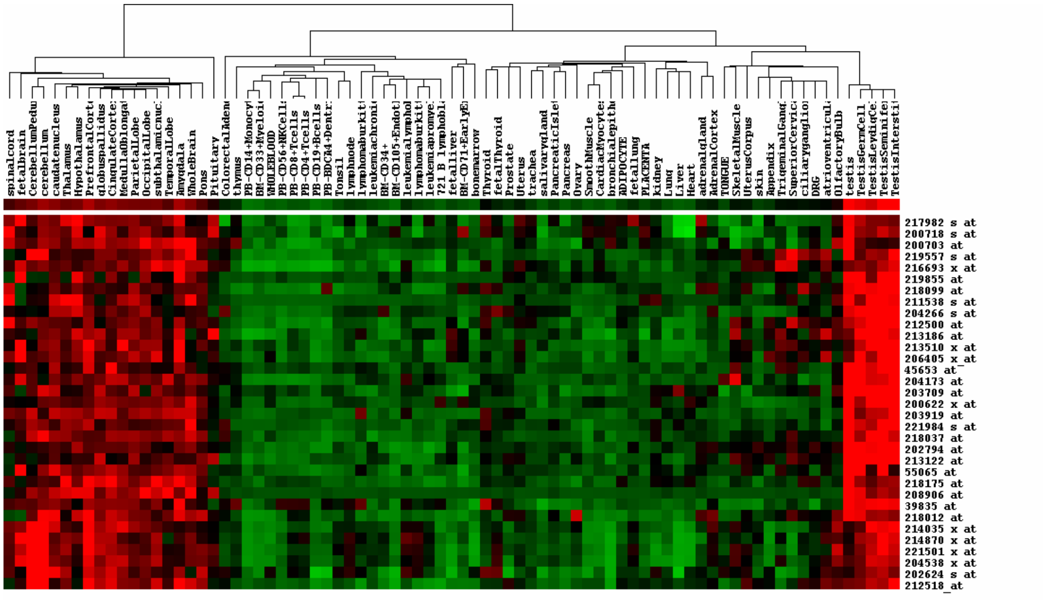

Additional figure 9. Enlarged image of the set of 33 genes that are expressed in neural testis-derived tissues (Marked with a blue line in figure 8).

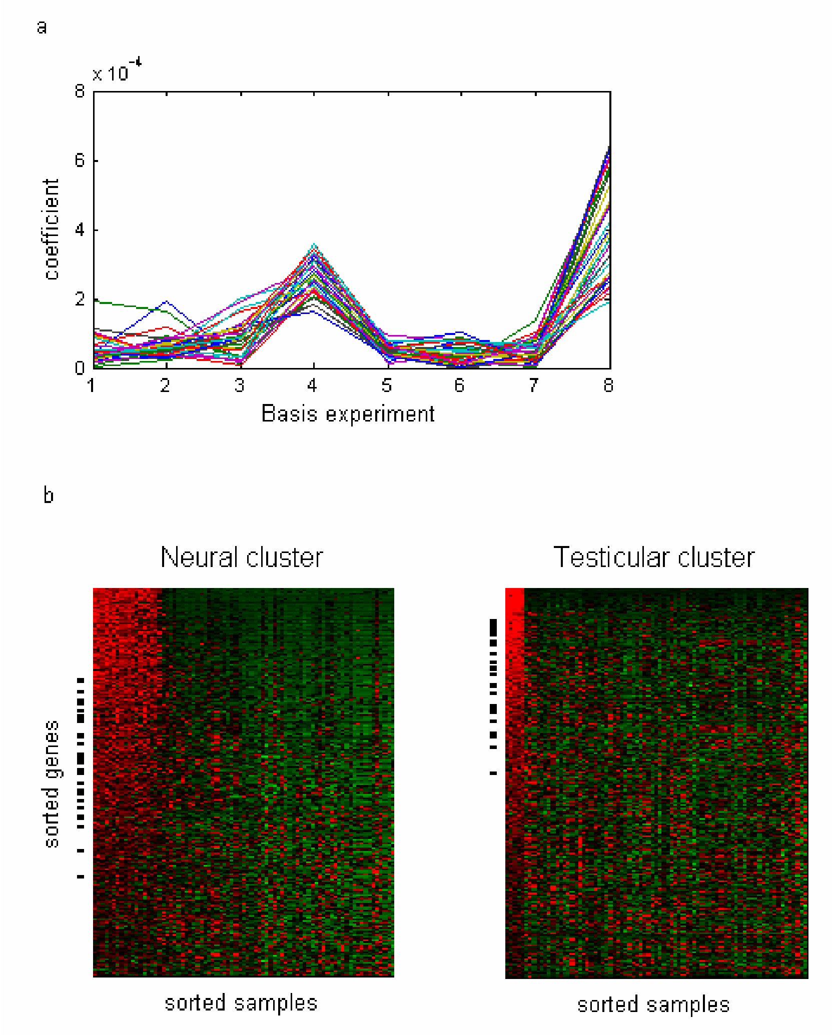

Additional figure 10. Example of genes associated to different gene modules. Different genes can be associated to different gene expression modules revealing that these genes can be expressed in different sets of experimental conditions. This is illustrated by 33 genes that showed a peak in the four and eight basis experiment (a). Each line represents the coefficients of a single gene in each column of W. (b) Sub-matrices containing genes and samples sorted by the fourth basis experiment and basis gene (neural feature) and the eight basis experiment and basis gene (testis feature). Only the top 3000 genes are shown. Common genes in both structures are marked with dash lines.
